# Functional studies of *Drosophila* zinc transporters reveal the mechanism for zinc excretion in Malpighian tubules

**DOI:** 10.1186/s12915-017-0355-9

**Published:** 2017-02-14

**Authors:** Sai Yin, Qiuhong Qin, Bing Zhou

**Affiliations:** State Key Laboratory of Membrane Biology, School of Life Sciences, Tsinghua University, Beijing, 100084 China

**Keywords:** Zinc homeostasis, *Drosophila*, Zinc excretion, Zinc reabsorption

## Abstract

**Background:**

Zinc is an essential metal involved in many physiological processes. Previous work has identified a set of zinc transporters involved in *Drosophila* dietary zinc absorption. However, zinc excretion and reabsorption, the other two important processes to maintain zinc homeostasis, are not as well understood. In this work, we screened all the potential zinc transporter Zip (SLC39) and ZnT (SLC30) members for their likely roles in zinc excretion in Malpighian tubules, an insect organ functionally analogous to mammalian kidneys.

**Results:**

Zip71B (CG10006, most homologous to hZIP5), in addition to the previously characterized ZnT35C (CG3994), was identified as being critical in zinc excretion. Tubule-specific knockdown of *Zip71B*/dZip5 reduces zinc accumulation in the tubules, but increases zinc levels in the body, resulting in survival defect under zinc excess conditions. Zip71B/dZip5 is localized to the plasma membrane at the basolateral side of the tubules, and is functionally epistatic to the apically localized ZnT35C in regulating the tubule zinc homeostasis. Our results indicate that Zip71B/dZip5 is involved in zinc import into the tubular cells from the circulation, and ZnT35C in turn effluxes the tubular zinc out. Notably, mammalian ZIP5, which is expressed in the kidney, functions analogously to Zip71B/dZip5 in the fly while hZIP4 cannot complement the loss of Zip71B/dZip5 function. Furthermore, *Zip71B/dZip5* expression is regulated by zinc so that, in response to toxic levels of zinc, the tubules can increase zinc efflux capability. We also characterized the role of dZnT1 (CG17723) in zinc reabsorption in Malpighian tubules. Finally, using a tubule calcification model, we were able to show that knockdown of *Zip71B/dZip5* or *ZnT35C* was able to mitigate stone formation, consistent with their roles in tubular zinc homeostasis.

**Conclusions:**

Our results start to sketch out a relatively complete picture of the zinc excretion process in *Drosophila* Malpighian tubules, and may provide a reference for relevant mammalian studies.

**Electronic supplementary material:**

The online version of this article (doi:10.1186/s12915-017-0355-9) contains supplementary material, which is available to authorized users.

## Background

Zinc is an essential micronutrient that serves as a catalytic or structural component of numerous enzymes and other proteins and accordingly contributes to a variety of fundamental biological processes [1–5]. Maintenance of zinc homeostasis is very important for human health. Either zinc deficiency or zinc toxicosis can lead to physiologic abnormalities [6–8]. Many common diseases including diabetes mellitus, Crohn’s disease, and Alzheimer’s disease are also linked with zinc dyshomeostasis [9–14].

Zinc homeostasis is tightly controlled by a series of transporter proteins that are broadly categorized into two major families, the Slc39a or Zip family and the Slc30a or ZnT family. Generally speaking, Zip proteins function in transporting zinc into the cytoplasm from extra- or intracellular vesicles, while ZnT proteins transport zinc in opposite orientations to efflux zinc out of the cytosol [15, 16]. Notably, some of these members also transport other metals like manganese, iron or cadmium, and sometimes in opposite directions [17–21]. In addition to these transporters, metallothioneins (MTs), a group of small, ubiquitous, cysteine-rich Zn-sensing molecules with high affinities for heavy metals, also play an essential role in zinc detoxification and in maintaining zinc homeostasis by buffering and storing intracellular zinc [22, 23]. The transcription of MT is regulated by metal-regulatory transcription factor 1 (MTF-1), which binds to the metal-response elements in the enhancer/promoter regions of MT genes [24–26].

At the organismal level, the physiological function of zinc transporters was shown to be essential for systemic zinc homeostasis and health [27, 28]. For example, in mammals, *ZnT1* knockout mice die at an embryonic stage, suggesting a key function for ZnT1 in fetal development [29]; *hZIP13* mutations have been shown to cause Ehlers–Danlos syndrome [30]; and human *ZIP4* has been found to be responsible for acrodermatitis enteropathica (AE), a human inherited disease caused by impaired zinc uptake [31–33].

*Drosophila* provides a valid model for studying metal homeostasis [34–41], and the core zinc transport and regulation machinery discovered in yeast and mammals is conserved in the fly [42–44]. The *Drosophila* genome encodes 10 Zip proteins and 7 ZnT proteins. In comparison, mammalian zinc transporters consist of 14 Zip members and 10 ZnT members. Phylogenetic analysis indicates that these proteins cluster into distinct subgroups [45]. *Drosophila* also has five MT genes, as well as the zinc-finger-containing transcription factor MTF-1 gene [46–48]. Molecular and cellular functions of *Drosophila* Zip/ZnT members were systematically analyzed via both over-expression and RNA interference using the GAL4-UAS system [45]. These zinc transporters were further analyzed based on their subcellular localizations and genetic interactions [49–51]. At the organismal level, a set of plasma membrane-resident zinc transporters was identified to participate in dietary zinc absorption. Zip42C.1 (CG9428), Zip42C.2 (CG9430) and Zip89B (CG6898) appear to be involved in uptaking zinc from the lumen into the enterocyte, and ZnT63C (CG17723) and ZnT77C (CG5130) work together to move absorbed zinc from the enterocyte into the circulatory system. The intracellular zinc transporters, such as ZnT86D (CG6672), were not directly involved in dietary zinc absorption [52–54].

Besides zinc absorption, zinc excretion and reabsorption are two other aspects to control body zinc homeostasis. Unfortunately, our understanding of these two important processes is still limited [55, 56]. *Drosophila* Malpighian tubules are renal organs that perform excretory functions like the kidney in vertebrates. The four tubules originate from the junction between the midgut and hindgut, and are packed as two pairs, with one pair migrating anteriorly and the other pair posteriorly [57–59]. The only player that was known to be involved in zinc excretion in Malpighian tubules is ZnT35C [60], which is homologous to a set of mammalian intracellular zinc transporters including ZnT2, 3, 4, and 8; it was not known what other players might be involved. The zinc exporter ZnT35C was found localized to the apical membrane of tubular cells and proposed to mediate zinc efflux from tubular cells. Interestingly, knocking down *ZnT35C* also significantly reduced kidney stone formation in a fly calcification model generated by inhibiting xanthine dehydrogenase (Xdh), suggesting that zinc homeostasis in tubules is important to this physiologic process [61].

Theoretically, there should be at least one zinc importer involved in zinc excretion in fly Malpighian tubules. In addition, from a physiological point of view, very likely one or more additional zinc transporters might be involved in zinc reabsorption, preventing the loss of this precious nutritional element under normal or low zinc conditions. In this study, we systematically studied the function of zinc transporters for their roles in zinc excretion in *Drosophila*. Our results identified Zip71B as a key player in zinc excretion and dZnT1 in zinc reabsorption. This study, together with previous work, began to reveal a primitive picture of players orchestrating the control of zinc excretion in *Drosophila* Malpighian tubules.

## Results

### Tubule-specific *Zip71B*-RNAi and *ZnT35C*-RNAi flies are sensitive to zinc

**Fig. 1:**
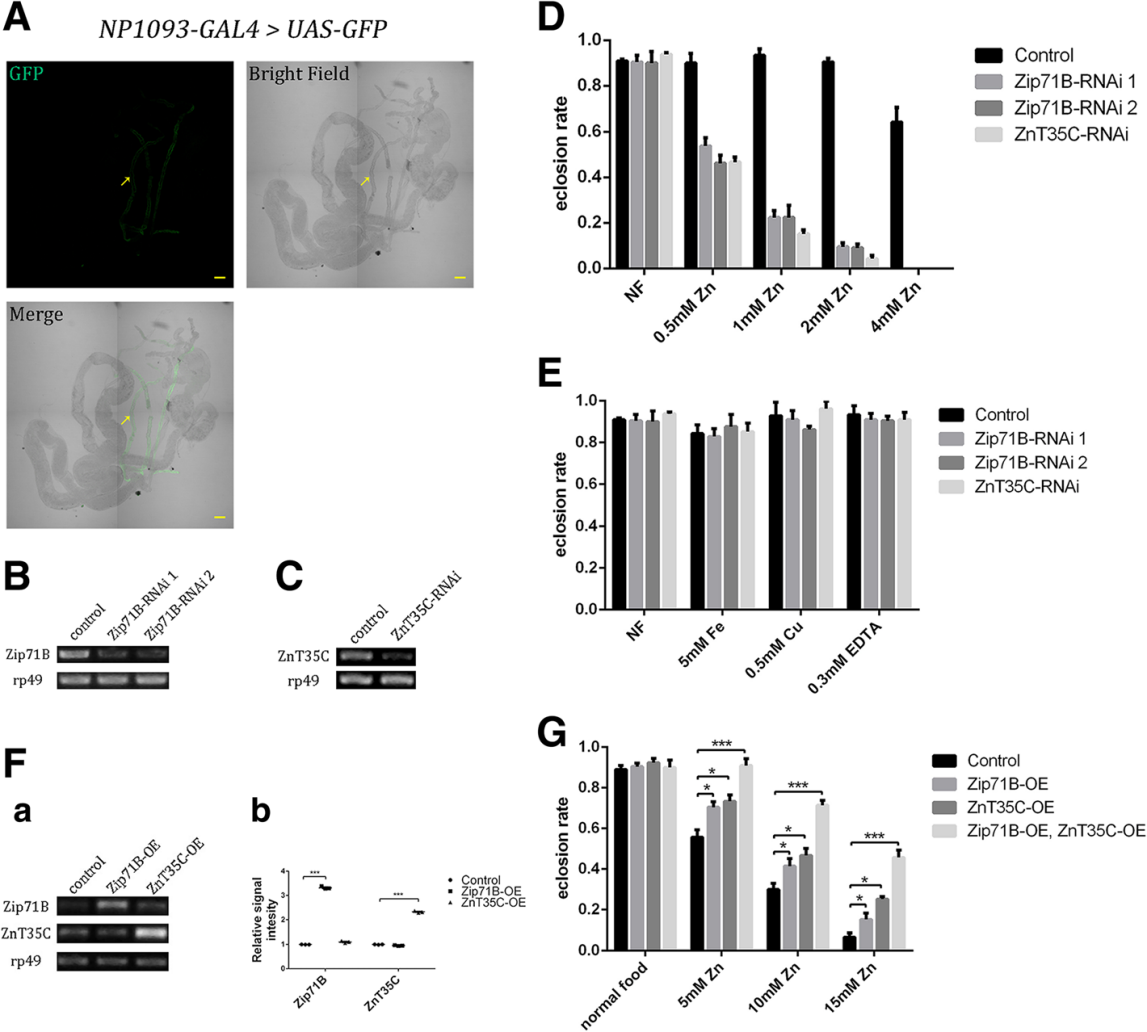
Tubule-specific modulation of *Zip71B* and *ZnT35C* expressions modifies flies’ zinc sensitivity. **A** Expression pattern of *NP1093-GAL4* > *UAS-GFP* in the gut of third-instar larvae. *NP1093-GAL4* is expressed specifically in the Malpighian tubules. Arrows denote the Malpighian tubules. Scale bars = 100 μm. **B, C** RT-PCR analysis of the knockdown effect of *Zip71B*-RNAi and *ZnT35C*-RNAi flies. *rp49* was used as internal control. **D, E** Survival rate of tubule-specific *Zip71B*-RNAi and *ZnT35C*-RNAi flies under different food conditions. Both *Zip71B*-RNAi and *ZnT35C*-RNAi flies show increased sensitivity toward zinc (means ± SEM, n = 6, *P* < 0.001; two-tailed Student’s *t* test), but not other metals or metal chelator EDTA. Genotypes of the flies are *NP1093-GAL4/+* for control, *Zip71B-RNAi/+; NP1093-GAL4/+* for *Zip71B*-RNAi, and *ZnT35C-RNAi/+; NP1093-GAL4/+* for *ZnT35C*-RNAi. **F** (**a**) RT-PCR analysis of the overexpression effect of *Zip71B*-OE and *ZnT35C*-OE flies. *rp49* was used as internal control. (**b**) Statistical analysis of RT-PCR result in (a); n = 3. Genotypes of the flies are *NP1093-GAL4/+* for control, *UAS-Zip71B/+; NP1093-GAL4/+* for *Zip71B*-OE, and *UAS-ZnT35C/+; NP1093-GAL4/+* for *ZnT35C*-OE. **G** Tubule-specific over-expression of Zip71B and ZnT35C confers resistance to high dietary zinc. While both *Zip71B*-OE and *ZnT35C*-OE flies showed resistance to high dietary zinc, *Zip71B-*OE and *ZnT35C-*OE flies displayed a synergistic effect. Data are presented as means ± SEM, n = 6. * *P* < 0.05, *** *P* < 0.001; two-tailed Student’s *t* test. Genotypes of the flies are *NP1093-GAL4/+* for control, *UAS-Zip71B/+; NP1093-GAL4/+* for *Zip71B*-OE, *UAS-ZnT35C/+; NP1093-GAL4/+* for *ZnT35C*-OE, and *UAS-ZnT35C/+; NP1093-GAL4/+* for *ZnT35C-*OE

*Drosophila* homologs of mammalian zinc transporters in the Zip and ZnT family include seven potential ZnT and ten Zip proteins [45]. Our previous work systematically analyzed *Drosophila* zinc transporters for their roles in dietary zinc absorption, enabling us to form a relatively clear picture of the zinc absorption [52, 53]. However, our knowledge is still limited for another important process, zinc excretion. To identify zinc transporters involved in zinc excretion, we used a Malpighian tubule-specific GAL4 line *NP1093* (Fig. 1a), crossed to RNAi lines of all potential Zip and ZnT members to knock down each of them, and tested sensitivities of the larvae to zinc overload. Among these zinc transporters, only *Zip71B*-RNAi and *ZnT35C*-RNAi flies are hypersensitive to zinc overload (Additional file 1: Table S1).

To confirm the sensitivity phenotype of *Zip71B-*RNAi is zinc specific, we analyzed the survival rate of *Zip71B*-RNAi flies on food with zinc or other metal addition. To reduce the possibility that the observed sensitivity arose from off-target effects of RNAi, we used two independent RNAi lines of *Zip71B*. On normal food, *Zip71B*-RNAi flies emerged with no aberrances compared to the control. However, as the concentration of zinc in the food increased, the survival rate of *Zip71B*-RNAi flies decreased dramatically; 4 mM zinc addition prevented almost all *Zip71B*-RNAi flies from surviving to the adult stage, suggesting that the *Zip71B-*RNAi flies are extremely sensitive to zinc overload. *ZnT35C*-RNAi flies also showed increased sensitivity to excess zinc, similar to *ZnT35C* mutant flies as reported before [60]. Iron, copper or zinc chelator EDTA addition has no obvious effects on *Zip71B-*RNAi or *ZnT35C-*RNAi flies, indicating that this sensitivity is specific to zinc overload (Fig. 1d, e).

Next, we examined the effects of tubule-specific overexpression of *Zip71B* on survival under excessive dietary zinc conditions. Consistently, overexpression of *Zip71B* confers resistance to high dietary zinc levels (5–15 mM zinc), similar to *ZnT35C* overexpression. Notably, overexpressing *Zip71B* and *ZnT35C* simultaneously enabled significantly stronger resistance to high levels of zinc, suggesting a synergistic effect in zinc excretion (Fig. 1g).

### Zinc is accumulated in the body when *Zip71B* is knocked down in the tubule

**Fig. 2:**
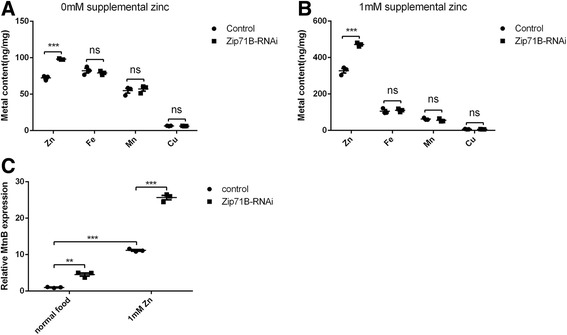
Zinc is accumulated in the body of *Zip71B*-RNAi fly. **a, b** Metal contents in *Zip71B*-RNAi larvae on normal and 1 mM zinc-supplemented food. Tubule-specific RNAi of *Zip71B* led to a significant increase in whole-body zinc content but no other metal content in both normal food and 1 mM zinc food. Metal content values represent three independent ICP-MS measurements and are normalized to dry body weight; data are presented as means ± SEM, n = 3. *** *P* < 0.001; two-tailed Student’s *t* test. **c** Real-time analysis showing up-regulation of *MtnB* in *Zip71B*-RNAi larvae on normal and 1 mM zinc-supplemented food. *rp49* was used as the reference gene for normalization. MtnB expression was shown as ratios relative to that of wild-type larvae raised on normal food. Values are presented as means ± SEM; n = 3. ** *P* < 0.01, *** *P* < 0.001, two-tailed Student’s *t* test. Genotypes of the flies are *NP1093-GAL4/+* for control and *Zip71B-RNAi/+; NP1093-GAL4/+* for *Zip71B*-RNAi

The above study showed that tubule-specific RNAi of *Zip71B* resulted in sensitivity to zinc overload. To analyze possible zinc homeostasis defects of *Zip71B*-RNAi flies, we measured total zinc content of the whole larvae using inductively coupled plasma-mass spectrometry (ICP-MS). Zinc content in *Zip71B-*RNAi flies increased dramatically compared to the control flies under either normal or zinc-supplement conditions. As control, levels of other metals including iron, copper and manganese were little changed (Fig. 2a, b). These results suggest that the sensitivity of *Zip71B*-RNAi larvae to excess zinc is due to failure of zinc excretion in the tubules, resulting in excessive zinc accumulation in the body.

MT B (*MtnB*) expression is sensitive to zinc increases in the cell. We thus preformed a real-time PCR analysis to test the transcription level of *MtnB* in *Zip71B*-RNAi and wild-type larvae. Compared to that in normal food, both wild-type and *Zip71B*-RNAi flies show a dramatic increase in *MtnB* transcription when reared on 1 mM zinc. In the normal food, the *MtnB* RNA level of *Zip71B-*RNAi larvae was about 400% of that of the normal wild-type, while in the presence of 1 mM zinc supplementation the *MtnB* RNA level of *Zip71B-*RNAi larvae was about 200% of that of the normal wild-type (Fig. 2c). This result is consistent with the notion that an increase of zinc level occurred in *Zip71B*-RNAi flies.

### Endogenous Zip71B localizes to the basolateral membrane of Malpighian tubule cells

**Fig. 3:**
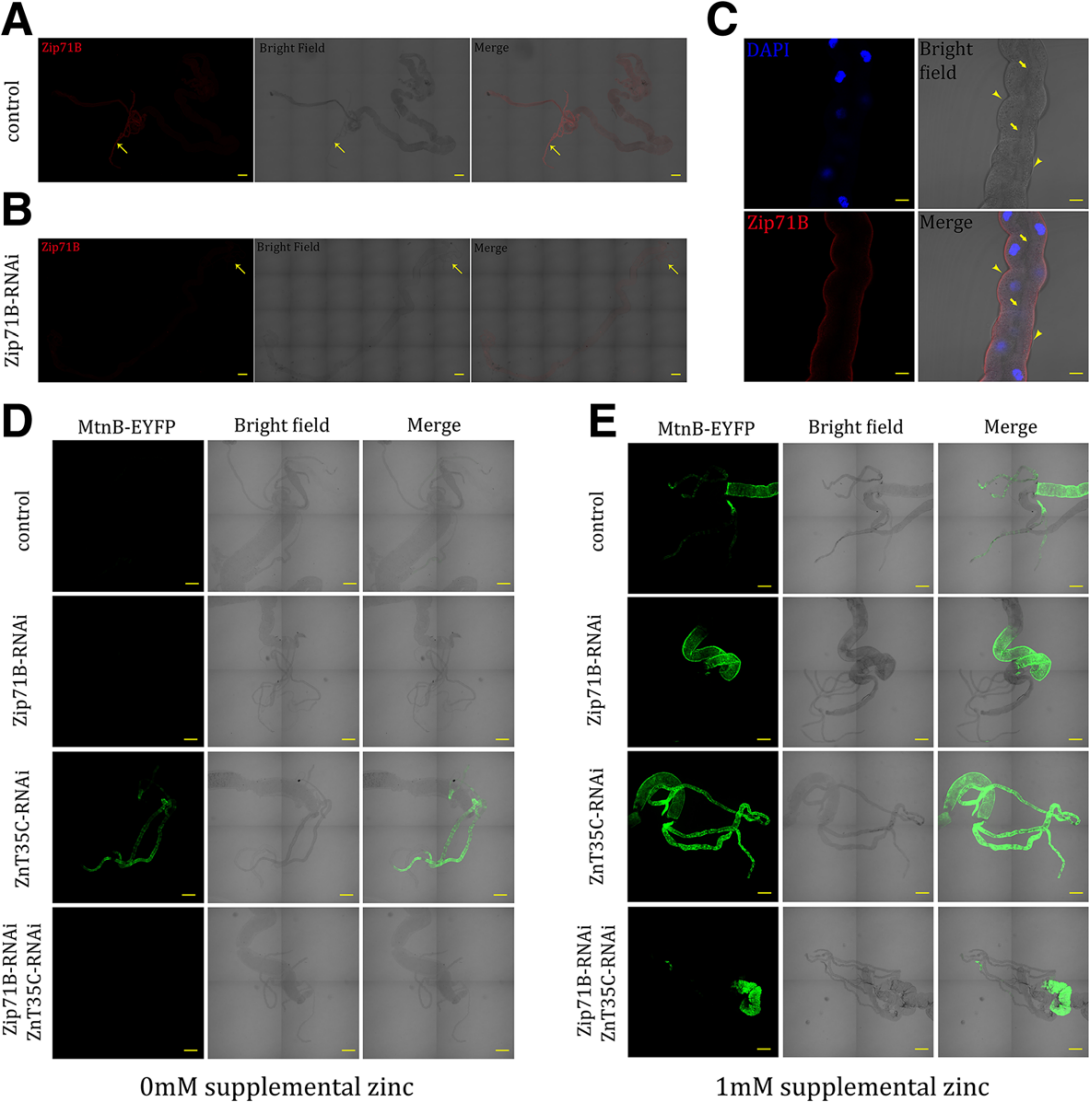
Zip71B is localized to the basolateral membrane to import zinc into the tubular cells. **a–c** Expression pattern and intracellular localization of Zip71B. Zip71B expression (immunofluorescence in red) in the Malpighian tubules of control larvae (**a**) and *Zip71B*-RNAi larvae (**b**) grown on normal food. Arrows denote Malpighian tubules. Scale bars = 100 μm. **c** Zip71B (red) is localized on plasma membrane and restricted to the basolateral side of tubule cells. DAPI (blue) shows the nucleus. Arrowheads indicate the basolateral membranes, and arrows indicate the apical membranes. Scale bars = 20 μm. Genotypes of the flies used are *NP1093-GAL4/+* for control and *Zip71B-RNAi/+; NP1093-GAL4/+* for *Zip71B*-RNAi. **d, e** *Zip71B* RNAi, in contrast to *ZnT35C* RNAi, resulted in zinc reduction in Malpighian tubules. Zinc changes are indicated by alterations of MtnB-EYFP fluorescence; (**d**) is on normal food and (**e**) is on zinc food. When reared on normal food, *Zip71B*-RNAi and *Zip71B, ZnT35C*-RNAi flies showed no obvious difference in fluorescence signal compared to controls, while *ZnT35C*-RNAi flies showed dramatically increased MtnB-EYFP fluorescence in Malpighian tubules. When reared on 1 mM zinc food. *ZnT35C*-RNAi flies showed further increased MtnB-EYFP fluorescence signal in tubules compared to control. In *Zip71B*-RNAi and *Zip71B, ZnT35C*-RNAi flies, MtnB-EYFP fluorescence was significantly decreased in tubules. Scale bars = 100 μm. Genotypes of the flies used are *MtnB-EYFP/+; NP1093-GAL4/+* for the control, *MtnB-EYFP/+; Zip71B-RNAi/+; NP1093-GAL4/+* for *Zip71B*-RNAi, *MtnB-EYFP/+; ZnT35C-RNAi/+; NP1093-GAL4/+* for *ZnT35C*-RNAi, and *MtnB-EYFP/+; Zip71B, ZnT35C-RNAi/+; NP1093-GAL4/+* for the double RNAi flies

According to FlyAtlas data [62], *Zip71B* transcript is mainly found in the Malpighian tubules. In order to examine its endogenous protein expression pattern, especially in subcellular locations, we generated an antibody against Zip71B. In dissected guts of wild-type third-instar larvae, Zip71B immunoreactivity was most prominent in the Malpighian tubules (Fig. 3a). The specificity of the antibody was confirmed by a significantly reduced staining in *Zip71B-*RNAi larvae (Fig. 3b).

The subcellular localization of endogenous Zip71B was revealed by immunofluorescence microscopy under high magnification. Zip71B was found to be localized on the plasma membrane and restricted to the basolateral side of the tubular cells (Fig. 3c). Considering that Zip71B is a Zip member, the basolateral distribution of Zip71B is consistent with its role in zinc excretion, i.e., absorbing zinc from circulation into the tubular cells.

Given that immunostaining of Zip71B also revealed slight expression in the intestine, implying a possible excretion role of Zip71B/dZip5 in the midgut, we additionally analyzed the roles of Zip71B/dZip5 and ZnT35C for zinc excretion in the intestine. To address this, we used a midgut-specific driver *NP3084. NP3084* predominantly activates expression in the midgut, with undetectable activity in the Malpighian tubes, as confirmed with *UAS-GFP* as the activity reporter (Additional file 2: Figure S1A). Knocking down *Zip71B/dZip5* or *ZnT35C* in the midgut made the flies slightly more susceptible to zinc overdose (Additional file 2: Figure S1B, C), suggesting that under zinc stress, the Malpighian tubes are the predominant organ for detoxification whereas the intestine likely plays an auxiliary role.

### Zip71B acts in concert with ZnT35C to move zinc across the tubular cells in zinc excretion

*MtnB-EYFP* transgene flies, wherein an enhanced yellow fluorescent protein (EYFP) cDNA is inserted behind the regulatory region of the zinc responsive gene *MtnB*, can be used as an indicator of intracellular zinc levels. *MtnB-EYFP* flies have been successfully used in monitoring zinc levels in the midgut constriction before [47, 52–54]. To analyze possible alterations of tubular zinc level in *ZnT35C-*RNAi and *Zip71B-*RNAi larvae, we crossed in the *MtnB-EYFP* transgene. Normal larvae raised on zinc-supplemented food show dramatically induced signal of MtnB-EYFP in both the tubules and posterior midgut, confirming that zinc accumulation in these tissues can be reflected by an increase of MtnB-EYFP fluorescence signal (Fig. 3d, e).

We then tested the MtnB-EYFP fluorescence signal in *ZnT35C-*RNAi and *Zip71B-*RNAi larvae. ZnT35C is reported to be localized to the apical membrane of Malpighian tubule cells, so our prediction is that knockdown of *ZnT35C* might accumulate zinc in tubule cells. Indeed, on both normal and zinc-supplemented food, *ZnT35C-*RNAi flies showed dramatically increased MtnB-EYFP fluorescence signal in tubules compared to the control (Fig. 3d, e), consistent with its role in moving zinc out from tubule cells to the lumen. In contrast, in *Zip71B-*RNAi flies, no significant change in MtnB-EYFP signal was seen in the normal food condition compared to the control (Fig. 3d, e). Further, when 1 mM zinc was supplemented in the diet, the MtnB-EYFP signal induction normally observed in the wild-type larvae was much less remarkable in the *Zip71B-*RNAi tubules, while the MtnB-EYFP signal intensity in the posterior midgut remains unchanged (Fig. 3e). These results indicate that zinc failed to be imported into the tubule cells in *Zip71B*-RNAi flies.

If Zip71B is responsible for zinc import into, and ZnT35C for zinc efflux out of, the tubule cells, we expect ZnT35C functions downstream of Zip71B. To confirm this epistatic relationship, we checked MtnB-EYFP signal in *Zip71B* and *ZnT35C* double RNAi animals. As expected, in the double RNAi larvae, MtnB-EYFP signal was significantly decreased in Malpighian tubules on zinc-supplemented food (Fig. 3e), similar to that in *Zip71B*-RNAi animals, indicating that Zip71B is indeed epistatic to ZnT35C. Taken together, we propose that Zip71B functions upstream of ZnT35C to transport zinc into tubule cells, and collaborates with ZnT35C to excrete zinc out of the body to maintain zinc homeostasis.

### *Zip71B* expression is regulated by dietary zinc

**Fig. 4:**
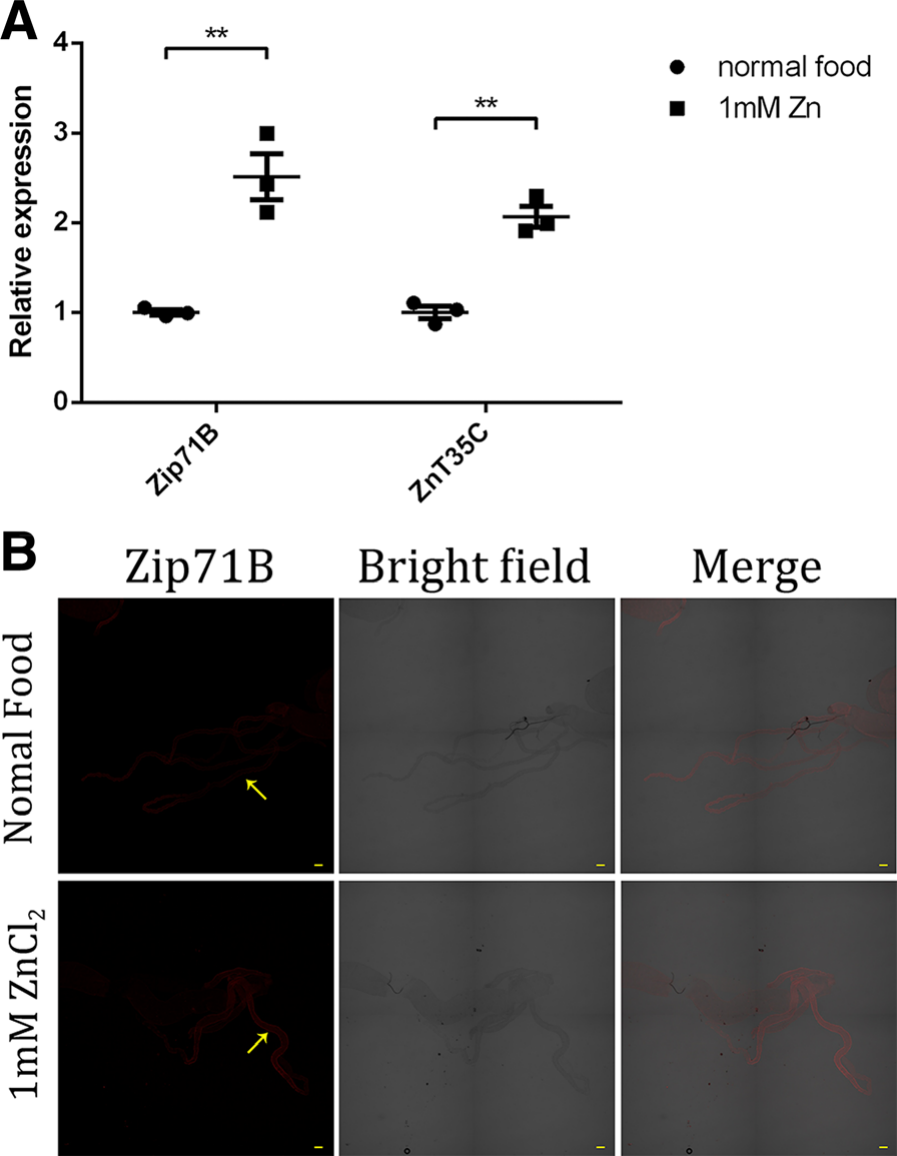
Both *Zip71B* and *ZnT35C* expressions are responsive to zinc. **a** Real-time analysis of mRNA levels of *Zip71B* and *ZnT35C* in dissected Malpighian tubules of wild-type animals cultured with normal food or 1 mM zinc-supplemented food. *Zip71B* was significantly induced by 1 mM zinc (** *P* < 0.01), as *ZnT35C* (** *P* < 0.01). Means ± SEM; n = 3, two-tailed Student’s *t* test. **b** Immunostaining reveals that Zip71B is upregulated by dietary zinc addition in Malpighian tubules (arrows). Scale bars = 50 μm

From a physiological point of view, zinc excretion activity increases in response to whole-body zinc stress. We therefore asked whether the expression of *Zip71B* in Malpighian tubules is subject to zinc regulation. We reared normal flies on standard and zinc supplemented diets, and examined the mRNA level of *Zip71B* in dissected Malpighian tubules using real-time PCR analysis. The expression levels of *Zip71B* were higher in flies fed zinc supplemented food, similar to that of the other zinc excretion gene *ZnT35C* (Fig. 4a). The zinc-inducibility of *Zip71B* expression was further confirmed by immunostaining in Malpighian tubules (Fig. 4b).

### Human ZIP5 can complement loss of Zip71B function

**Fig. 5:**
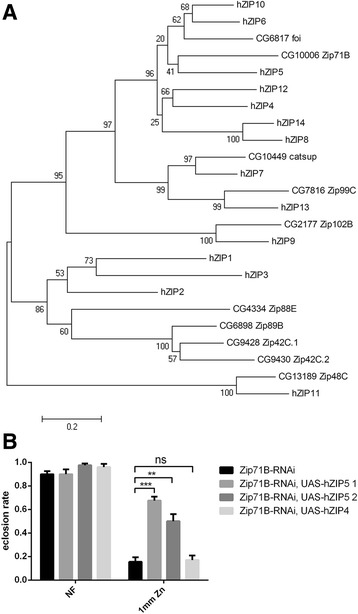
*Zip71B* loss of function can be complemented by *hZIP5* but not *hZIP4*. **a** Phylogenetic tree revealing the relationship among human and *Drosophila* Zip members. Tree was generated and displayed by using MEGA software version 4. Zip71B shares the highest homology with human ZIP5. **b** *hZIP5* expression can rescue the sensitivity of *Zip71B*-RNAi larvae on food supplemented with 1 mM Zinc. Data are presented as means ± SEM; n = 6. ** *P* < 0.01, *** *P* < 0.001, two-tailed Student’s *t* test. Genotypes of the flies used are *UAS-EGFP/+; Zip71B-RNAi/+; NP1093-GAL4/+* (*Zip71B*-RNAi fly), *UAS-hZIP5/+; Zip71B-RNAi/+; NP1093-GAL4/+* (*Zip71B*-RNAi, *UAS-hZIP5* fly), and *UAS-hZIP4/+; Zip71B-RNAi/+; NP1093-GAL4/+* (*Zip71B*-RNAi, *UAS-hZIP4* fly)

The protein encoded by *Zip71B* shares the highest homology with human ZIP5 (32% identity and 48% similarity; Fig. 5a). Interestingly, rodent ZIP5 (mZIP5) was also found to be localized to the basolateral membrane of mouse intestinal enterocytes where it plays an important role in zinc excretion [55]. To test whether hZIP5 can functionally rescue the zinc sensitive phenotype of *Zip71B-*RNAi flies, we generated *UAS*-*hZIP5* transgenic flies, introduced the transgene by crossing them with *Zip71B*-RNAi flies, and used the *NP1093-GAL4* line to drive the tubular expression. As shown in Fig. 5b, expression of hZIP5 can partially but significantly rescue the survival of *Zip71B*-RNAi flies on zinc-supplemented food, while as control, expression of hZIP4 showed no obvious rescue effect. These results suggest Zip71B is not only most homologous to mammalian ZIP5 in protein sequence, but functions analogously. We thus propose Zip71B as dZip5.

### dZnT1 functions in zinc reabsorption in *Drosophila* Malpighian tubules

**Fig. 6:**
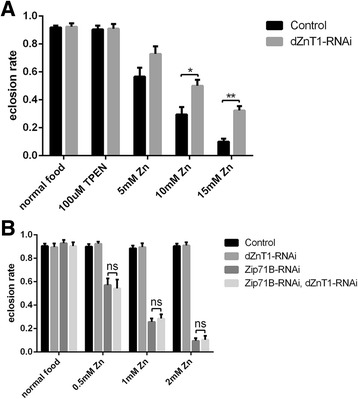
Tubule-specific *dZnT1* RNAi flies display resistance to high dietary zinc. **a** Survival rate of *dZnT1*-RNAi flies on different food conditions. There is no effect on survival of *dZnT1*-RNAi flies on normal and zinc-chelated (100 μM TPEN) food compared to control flies. However, *dZnT1*-RNAi flies survived significantly better on high dietary zinc (10 and 15 mM zinc) compared to wild-type flies. **b** Knocking down *dZnT1* in the tubule could not suppress zinc sensitivity of *Zip71B*-RNAi flies. Data are presented as means ± SEM; n = 6. * *P* < 0.05, ** *P* < 0.01, two-tailed Student’s *t* test. Genotypes of the flies used are *NP1093-GAL4/+* (control fly), *dZnT1-RNAi/+; NP1093-GAL4/+* (*dZnT1-RNAi* fly), *Zip71B-RNAi/+; NP1093-GAL4/+* (*Zip71B*-RNAi fly) and *dZnT1-RNAi/Zip71B-RNAi; NP1093-GAL4/+* (*Zip71B, dZnT1*-RNAi fly)

Zinc reabsorption is another important process for zinc homeostasis; however, it remains unknown which zinc transporter is involved in zinc reabsorption in Malpighian tubules. dZnT1 was reported to be localized on the basolateral membrane of Malpighian tubule cells. This distribution pattern of dZnT1 suggested its role in zinc reabsorption [52]. To verify this hypothesis, we examined the survival rate of *dZnT1-RNAi* flies driven by *NP1093-GAL4*. While *dZnT1*-RNAi flies showed no obvious defect on normal food and zinc-chelated food, they were much more resistant to high levels of dietary zinc (such as 10–15 mM) (Fig. 6a).

To investigate the genetic interaction between dZnT1 and Zip71B (dZip5) in Malpighian tubules, we made *dZnT1, Zip71B* double RNAi flies by recombining them together. Since *dZnT1*-RNAi flies are more resistant to zinc overload presumably due to reduced zinc reabsorption, we asked whether knockdown of dZnT1 in tubules could rescue the zinc sensitivity phenotype of *Zip71B*-RNAi flies. The survival assay on 1 mM zinc-supplemented food showed that the double RNAi flies are still sensitive to excess zinc, to a similar extent as the *Zip71B*-RNAi flies (Fig. 6b). These results indicate that Zip71B (dZip5) is epistatic to dZnT1, and in this case, dZnT1 functions downstream of Zip71B/dZip5 in tubules for zinc recovery from excretion.

### Tubule-specific inhibition of *Zip71B (dZip5)* and *ZnT35C* suppresses the formation of tubule stones caused by inhibition of xanthine dehydroxylase

**Fig. 7:**
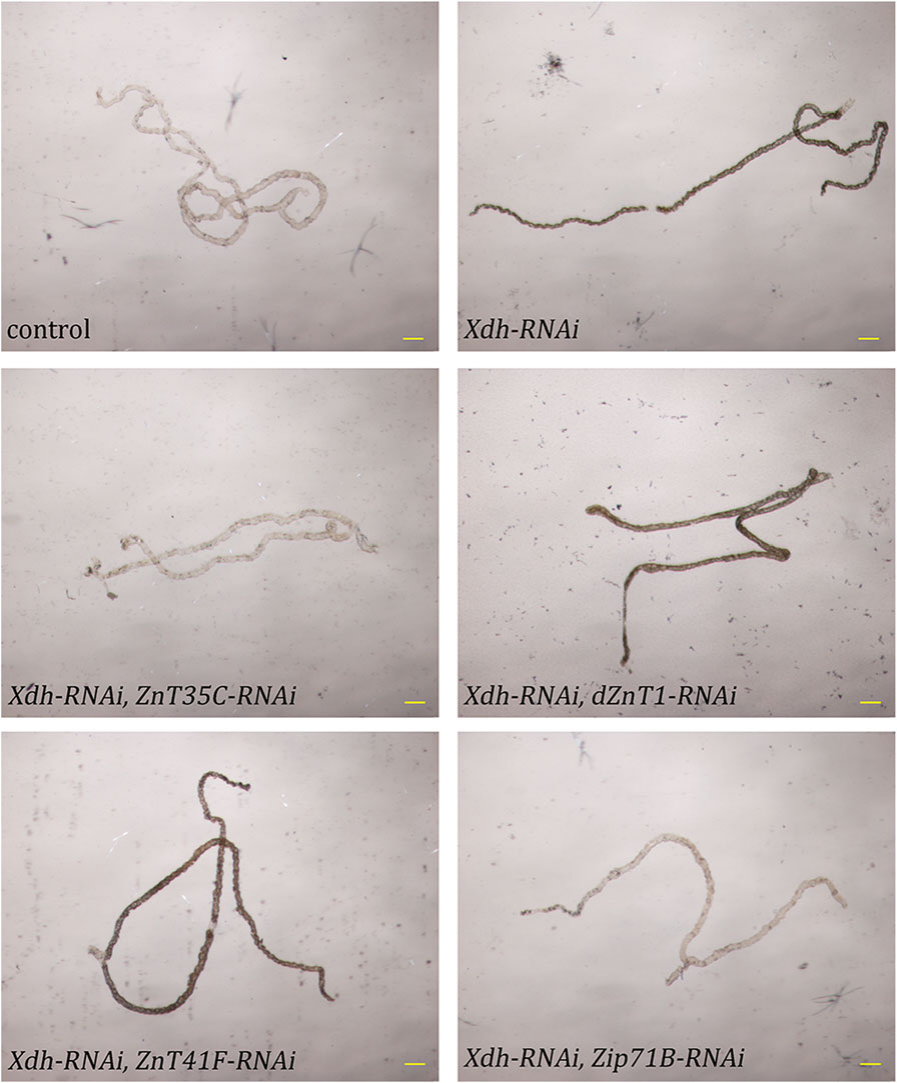
Tubule-specific inhibition of *Zip71B* and *ZnT35C* suppresses the formation of tubule stones. Representative tubule images taken from different groups of flies. Scale bars = 200 μm. Tubule-specific RNAi of *Xdh* resulted in significantly increased tubule stone formation compared to controls. Knockdown of *Zip71B* or *ZnT35C* in Malpighian tubules significantly suppressed the stone formation, while knockdown of *dZnT1* or *ZnT41F* displayed no obvious fly stone reduction. Genotypes of the flies are *NP1093-GAL4/+* (control fly), *Xdh-RNAi/+; NP1093-GAL4/+* (*Xdh*-RNAi fly), *ZnT35C-RNAi/+; Xdh-RNAi/+; NP1093-GAL4/+* (*Xdh*-RNAi *ZnT35C-*RNAi fly), *dZnT1-RNAi/+; Xdh-RNAi/+; NP1093-GAL4/+* (*Xdh*-RNAi *dZnT1-*RNAi fly), *ZnT41F-RNAi/+; Xdh-RNAi/+; NP1093-GAL4/+* (*Xdh*-RNAi *ZnT41F-*RNAi fly), *Zip71B-RNAi/+; Xdh-RNAi/+; NP1093-GAL4/+* (*Xdh*-RNAi *Zip71B-*RNAi fly)

A recent work showed that zinc metabolism in Malpighian tubules is related to ‘kidney stone’ formation in *Drosophila*; a reduction of the zinc level in the tubular lumen led to decreased stone formation. Three ZnT members (ZnT35C, dZnT1, and ZnT41F), when ubiquitously knocked down, were identified to be involved in this process [61]. We used tubule-specific *NP1093* instead of the original ubiquitous GAL4 line to knock down these genes, and found that tubule-specific suppression of *ZnT35C*, but not *dZnT1* and *ZnT41F*, affected tubular stone formation. These results suggest that *dZnT1* and *ZnT41F* may mediate tubular zinc homeostasis mostly in an indirect way. dZnT1, for example, is a key protein for dietary zinc absorption in addition to its role in the tubular zinc reabsorption. Consistent with its essential role in the tubular zinc homeostasis, tubule-specific knockdown of *Zip71B(dZip5)* also suppressed the stone formation (Fig. 7).

## Discussion

Zinc homeostasis in *Drosophila* is controlled by multiple biological processes. In this work, we focused on zinc excretion in *Drosophila* Malpighian tubules. Using the tubule-specific GAL4 line *NP1093*, we knocked down all the ten Zip members and seven ZnT members individually to screen for the zinc transporters participating in zinc excretion in Malpighian tubules. Zip71B/dZip5 was found to be involved in this process. *Zip71B-RNAi* flies displayed no developmental aberrance on normal media; however, knockdown of *Zip71B/dZip5* led to hypersensitivity to zinc overload, similar to *ZnT35C*-*RNAi* flies. Fine analysis revealed that, in contrast to *ZnT35C* RNAi, knockdown of *Zip71B/dZip5* resulted in decreased zinc content in the tubules. We concluded that Zip71B/dZip5 acts upstream of ZnT35C to drive zinc excretion from the body, while dZnT1 fulfills the task of zinc reabsorption. All three players work together to regulate the zinc excretory process in the Malpighian tubules.

It is quite remarkable that Zip71B is most homologous to hZIP5, and hZIP5 can partially substitute *Drosophila* Zip71B’s function in Malpighian tubules while hZIP4 cannot. *mZIP5* has relatively high expression level in mouse kidneys [63], just like Zip71B in the Malpighian tubules. Why can hZIP4 not rescue dZip71B/dZip5, given that both are zinc importers? We think this is likely due to the fact that Zip71B/dZip5 has to be located to the basolateral side of the tubule cells to function properly, and hZIP4 may lack the proper signal sequence or structure to be targeted there. If a zinc importer is not correctly positioned, it is hard to fulfill its task of unidirectional mobilization of zinc from the circulation to the tubular cells.

Malpighian tubules, affiliated with the intestine, are renal organs of *Drosophila* that perform excretory and osmoregulatory functions, similar to kidney in vertebrates and mammals. In zinc excretion studies conducted in mammals, the results of isotropic tracer studies suggest that most zinc is excreted through intestinal cells under normal zinc conditions [64], and mZIP5 is involved in this process [55]. Similar results were also found in *C. elegans*; *ttm-1*, one of the Cation Diffusion Facilitator family members, was shown to work in intestinal cells to promote zinc excretion, protecting animals from zinc toxicity [56]. In *Drosophila*, we found that main zinc excretion occurs in Malpighian tubules. Why this difference? In our experiment, zinc excretion was functionally analyzed under zinc excess or toxic conditions. It is possible that under normal conditions, most zinc excreted will be re-absorbed by the tubules; in this case, little will be really excreted, similar to what was found in mammalian zinc isotope studies. Notably, ZnT35C and Zip71B/dZip5 are also expressed in *Drosophila* midgut, and functionally participate in zinc excretion as midgut-specific knockdown of these two genes resulted in sensitivity to zinc excess, albeit to a lesser extent. In light of this, we speculate that, under normal conditions, more zinc might be excreted in the midgut while zinc is mostly reabsorbed in the Malpighian tubules; and when zinc is in excess, most zinc is detoxified through the Malpighian tubules; this might also happen in mammals. This notion is supported by the observation that whole body knockout of *mZIP5* displays additional zinc accumulation in the liver, compared to intestine-specific knockout of *mZIP5* [55], suggesting intestine is not the only important zinc excretion route in mammals. It is possible that mZIP5, as a zinc importer, is involved in mouse kidney zinc excretion. Therefore, current data suggests a somewhat similar process of zinc excretion and control may occur in mammals and the fly. Proteins playing analogous roles to *Drosophila* ZnT35C and dZnT1 during zinc excretion/reabsorption in mammalian kidneys remain to be identified.

How regulation of Zip5 and Zip71B/dZip5 expressions is achieved might be a complex story, and not yet well characterized. It has been reported that mZIP5 gets internalized and degraded under zinc deficiency conditions [63]. Burke’s lab has reported that Zip71B/dZip5 is a highly efficient transporter, much more potent than other Zip members; it is the only Zip protein that was found to cause zinc toxicity phenotype in eyes when expressed alone [45, 49, 50]. The absence of any detectable Zip71B^eGFP^ signal in larval salivary glands was interpreted by the fact that Zip71B/dZip5 might be down-regulated at the protein level to prevent zinc toxicity caused by excess zinc uptake. We found that *Zip71B/dZip5* responds to zinc excess in the Malpighian tubules by an increase in transcription. It is possible that *Zip5/Zip71B* expression is differentially regulated by zinc in different tissues. Despite this difference, they are alternative strategies to achieve the same goal, i.e., to effectively and economically regulate zinc transport in response to zinc levels or body zinc needs.

## Conclusions

**Fig. 8:**
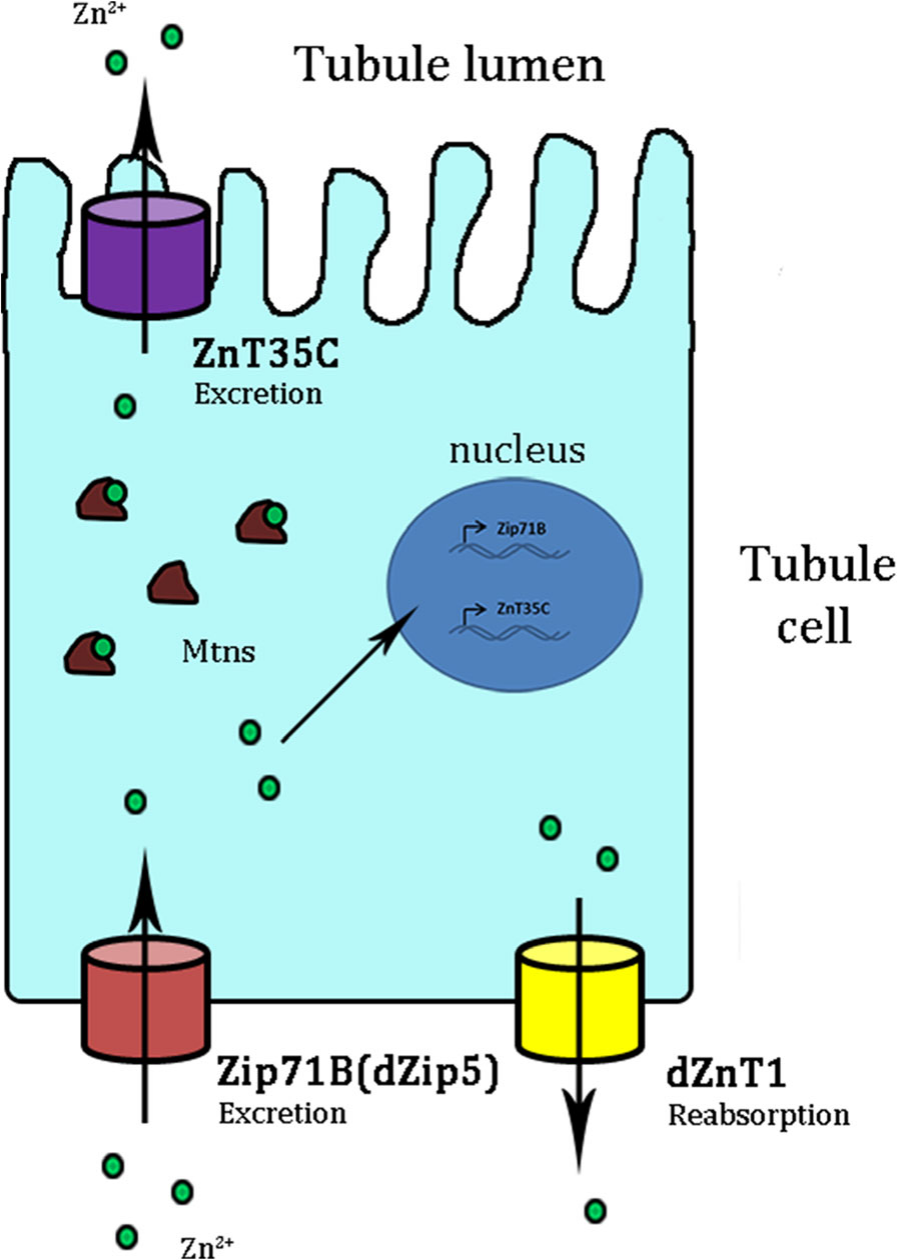
A model for zinc excretion in *Drosophila* Malpighian tubules. Zinc homeostasis in *Drosophila* Malpighian tubules is mediated by a set of plasma membrane-resident zinc transporters. Zip71B (dZip5) is localized to the basolateral membrane of tubule cells and responsible for zinc import from the body into Malpighian tubules. ZnT35C is localized to the apical membrane of tubule cells and pumps zinc out into tubule lumen. Zip71B/dZip5 and ZnT35C act in concert for moving zinc across the tubule cells in zinc excretion. dZnT1 is localized to the basolateral membrane of tubule cells and functions in zinc reabsorption to recover zinc from excretion. The transcription of *Zip71B/dZip5* and *ZnT35C* is subject to the regulation of zinc ion

Zinc excretion and reabsorption are important processes for whole-body zinc homeostasis control; however, the mechanisms of these controls are not well understood. *Drosophila* Malpighian tubules, functionally similar to mammalian kidneys, offer a model to study this intricate process. By systematically analyzing the roles of zinc transporters in *Drosophila* zinc excretion processes, we found that Zip71B (CG10006)/dZip5, which is localized to the basolateral membrane of tubule cells, functions in transporting zinc from body into Malpighian tubules, and acts in concert with apically resident ZnT35C (CG3994) to pump zinc out of the body to maintain zinc homeostasis. Both Zip71B/dZip5 and ZnT35C are subject to zinc regulation. dZnT1 (CG17723), on the other hand, is responsible for zinc reabsorption in Malpighian tubules. This study helps sketch out, for the first time in animals, a model of zinc excretion process (Fig. 8). These findings will be helpful for zinc excretion research in general, and provide a reference for future understanding of zinc metabolism in higher animals.

## Methods

### Plasmid

UAS-*Zip71B* and UAS-*ZnT35C* were generated by PCR amplification of the coding region of *Zip71B* and *ZnT35C* from *Drosophila* cDNA, and cloned into the vector pUAST. UAS-*hZIP5* and UAS-*hZIP4* were generated by PCR amplification of the coding region of *hZIP5* and *hZIP4* from human liver cDNA (Gibco BRL, Grand Island, NY, USA) and cloned into pUAST. All the constructs were verified by sequencing.

### Fly stocks, culture media, and transgenics

Fly stocks were raised at 18 °C, and all the experiments were performed at 25 °C on standard cornmeal food. When necessary, the food was supplemented with metals or metal chelators at stated concentrations. Fly stocks *act-GAL4/CyO* (Bloomington #4414), *da-GAL4* (Bloomington #8641) and *UAS-EGFP* (Bloomington#5130) were obtained from the Bloomington Stock Center (Bloomington, IN, USA); *NP3084* (DGRC# 113094) and *NP1093* (DGRC# 103880) lines were obtained from the *Drosophila* Genetic Resource Center at the Kyoto Institute of Technology (Kyoto, Japan). The *MtnB-EYFP* line was a kind gift from Dr. Walter Schaffner (University of Zurich, Zurich, Switzerland). The RNAi lines were provided by the VDRC (Vienna, Austria) or custom made at the Tsinghua Fly Center (Beijing, China), Zip71B-RNAi (VDRC#44539 and TH00155.N), ZnT35C-RNAi (VDRC#103263 and TH00119.N), ZnT41F-RNAi (VDRC#5390), and Xdh-RNAi (THU5626). Transgenic flies were generated in *w* ^*1118*^ background by P-element-mediated transformation.

### Fly eclosion assays

*NP1093* or *NP3084* homozygous flies were crossed with different transgenic flies, and the progeny were reared on food containing different metals or metal chelators, as indicated in each experiment. The density of each vial was controlled to 70 larvae, and the total number of emerged adults of each genotype was counted.

### Metal content assay

Flies were reared on normal food or food supplemented with 1 mM zinc from eggs until late third-instar larval stage. About 30 larvae were collected, weighed, and dissolved in 1 mL 65% HNO_3_, boiled in 100 °C water bath for 8 min, and diluted to 10 mL for metal content analysis with ICP-MS (IRIS Intrepid II XSP, Thermo Electron Corporation, Waltham, MA, USA). Data were collected and analyzed using the native software (ICP Expert).

### RNA isolation, semi-quantitative RT-PCR, and quantitative real-time PCR

Total RNA was extracted from adults, larvae or Malpighian tubules from third-instar larvae using the TRIzol reagent (Invitrogen, Carlsbad, CA, USA). cDNA was reverse-transcribed from 1 μg total RNA with TransScript First-Strand cDNA Synthesis SuperMix (TransGen Biotech Co., Ltd., Beijing, China), in accordance with the manufacturer’s instructions.

Semi-quantitative RT-PCR was performed using the specific primers corresponding to partial regions of the analyzed genes. Intensity of gel bands was quantitated using ImageJ densitometry. Real-time PCR was performed on StepOnePlusTM (Life Technologies, USA) using SYBR Green PCR Master Mix (Applied Biosystems). Fold change was determined by comparing target gene expression with the reference gene expression (*rp49*) under the same conditions. The primers used for PCR were: *Zip71B*, GACCGCTCACCCCAGTAG and ACCCACCGACATGCCCAC; *ZnT35C*, GTGTAAGCGGTATCAGAACTC and CTCCCAACTATATGCACTACG; *mtnB*: ATCAGTTCGCCTCAGCCAAG and GCAAACGCACTGGCAATCCT; *rp49*: GCACCAAGCACTTCATCC and CGATCTCGCCGCAGTAAA.

### Antiserum preparation

Rabbit polyclonal antibody was raised against His-Zip71B fusion protein. Briefly, the cDNA fragment encoding the N-terminal 240 aa of Zip71B was synthesized and cloned into pQE80L vector (Qiagen, Hilden, Germany). The recombinant protein was purified and injected into New Zealand White rabbits for antibody generation.

### Immunohistochemistry and microscopy

For immunohistochemistry, antibody for Zip71B was preabsorbed with fixed *w* ^*1118*^ embryos before performing staining on fly tissues. Third-instar larvae were collected and dissected in phosphate-buffered saline (PBS), fixed with paraformaldehyde and stained following standard procedures. Anti-Zip71B were used (1:250 dilution) in combination with TRITC -conjugated goat anti-rabbit IgG (ZF-0316, Zhongshan Goldenbridge Biotechnology Co. Ltd., Beijing, China). For nuclear staining, samples were incubated in 50 ng/mL DAPI for 10 min and washed three times in PBS. Slides were mounted with 50% glycerol/PBS.

The immunostainings of Zip71B were examined using a Zeiss LSM710 Meta confocal microscope (Zeiss, Germany). The fluorescence of MtnB-EYFP was captured with an ECLIPSE 80i microscope attached to a DXM1200F digital camera (both Nikon, Tokyo, Japan).

### Statistical analysis

All data were analyzed by Student’s *t*-test using GraphPad Prism version 6.00 for Windows (GraphPad Software, La Jolla California USA). Statistical results were presented as means ± SEM. Asterisks indicate critical levels of significance (* *P* < 0.05, ** *P* < 0.01, and *** *P* < 0.001).

## Additional files

Additional file 1: Table S1. Phenotype screening of RNAi lines covering all the 10 ZnT and 7 Zip members for zinc excretion in the Malpighian tubules. Expression was driven by tubule-specific GAL4, *NP1093*. (TIF 319 kb)
Additional file 2: Figure S1. Gut-specific *Zip71B* and *ZnT35C* RNAi flies display only slight sensitivity to zinc overload. (A) Expression pattern of *UAS-GFP* driven by *NP3084-*GAL4 line was visualized in dissected third-instar larvae. *NP3084* directs expression all over the midgut, but not in the Malpighian tubules. The yellow arrow denotes the Malpighian tubules, arrowhead denotes the hindgut, and star denotes the midgut. Scale bars = 100 μm. (B, C) Survival rate of gut-specific *Zip71B*-*RNAi* and *ZnT35C*-*RNAi* flies under different food conditions (means ± SEM, n = 6). Genotypes of the flies are *NP3084-GAL4/+* for control, *Zip71B-RNAi/+; NP3084-GAL4/+* for *Zip71B*-RNAi, and *ZnT35C-RNAi/+; NP3084-GAL4/+* for *ZnT35C*-RNAi. (TIF 890 kb)
Additional file 3: Table S2. Original data for RT-PCR and metal content analyses. (XLSX 13 kb)
