# Supplementary figures and images for: Functional studies of Drosophila zinc transporters reveal the mechanism for zinc excretion in Malpighian tubules
Source: BMC Biol. 2017 Feb 14;15:12. doi: 10.1186/s12915-017-0355-9 (PMC5309981; doi:10.1186/s12915-017-0355-9)

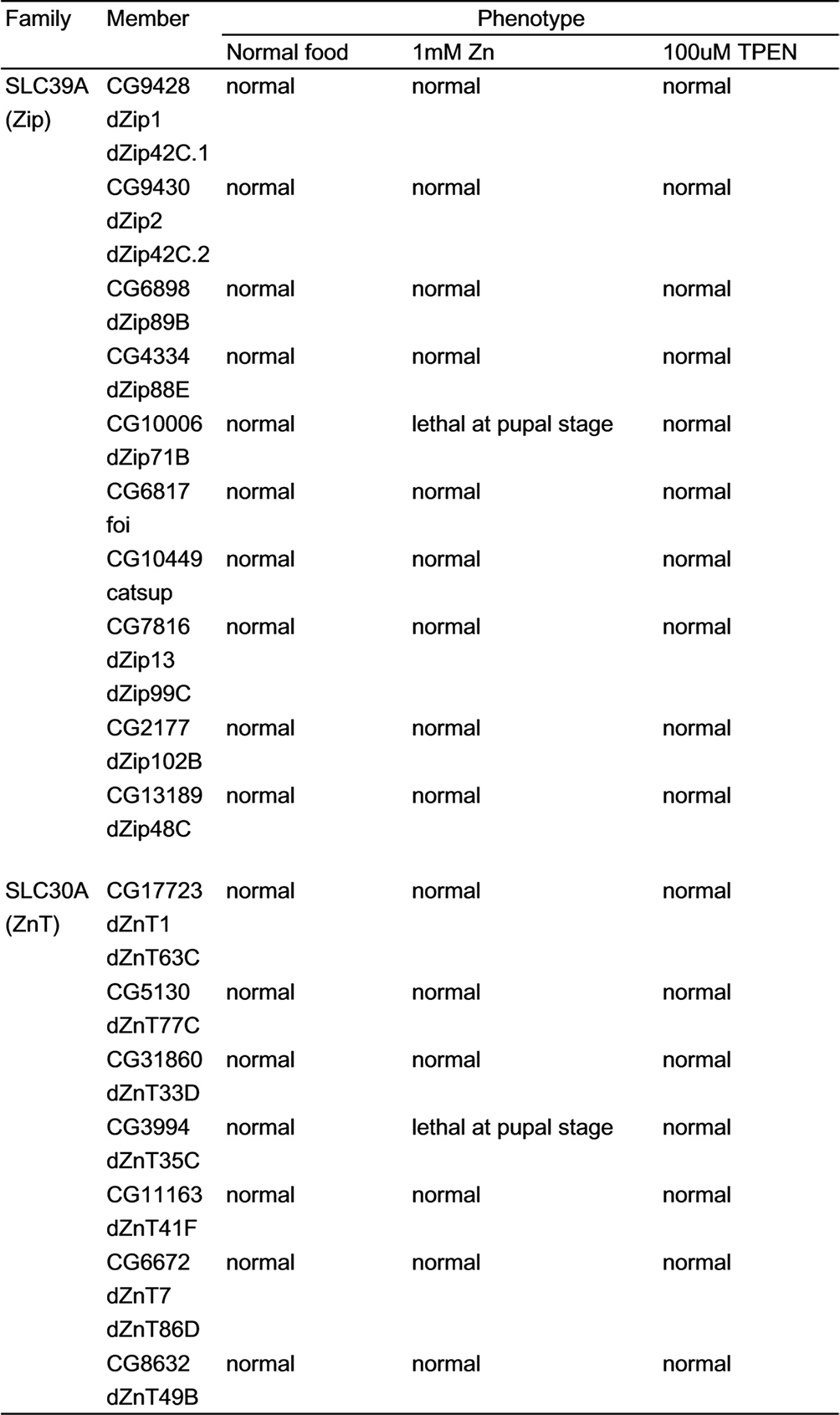

Supplement: Additional file 1: Table S1. — Phenotype screening of RNAi lines covering all the 10 ZnT and 7 Zip members for zinc excretion in the Malpighian tubules. Expression was driven by tubule-specific GAL4, NP1093. (TIF 319 kb) [file 12915_2017_355_MOESM1_ESM.tif]

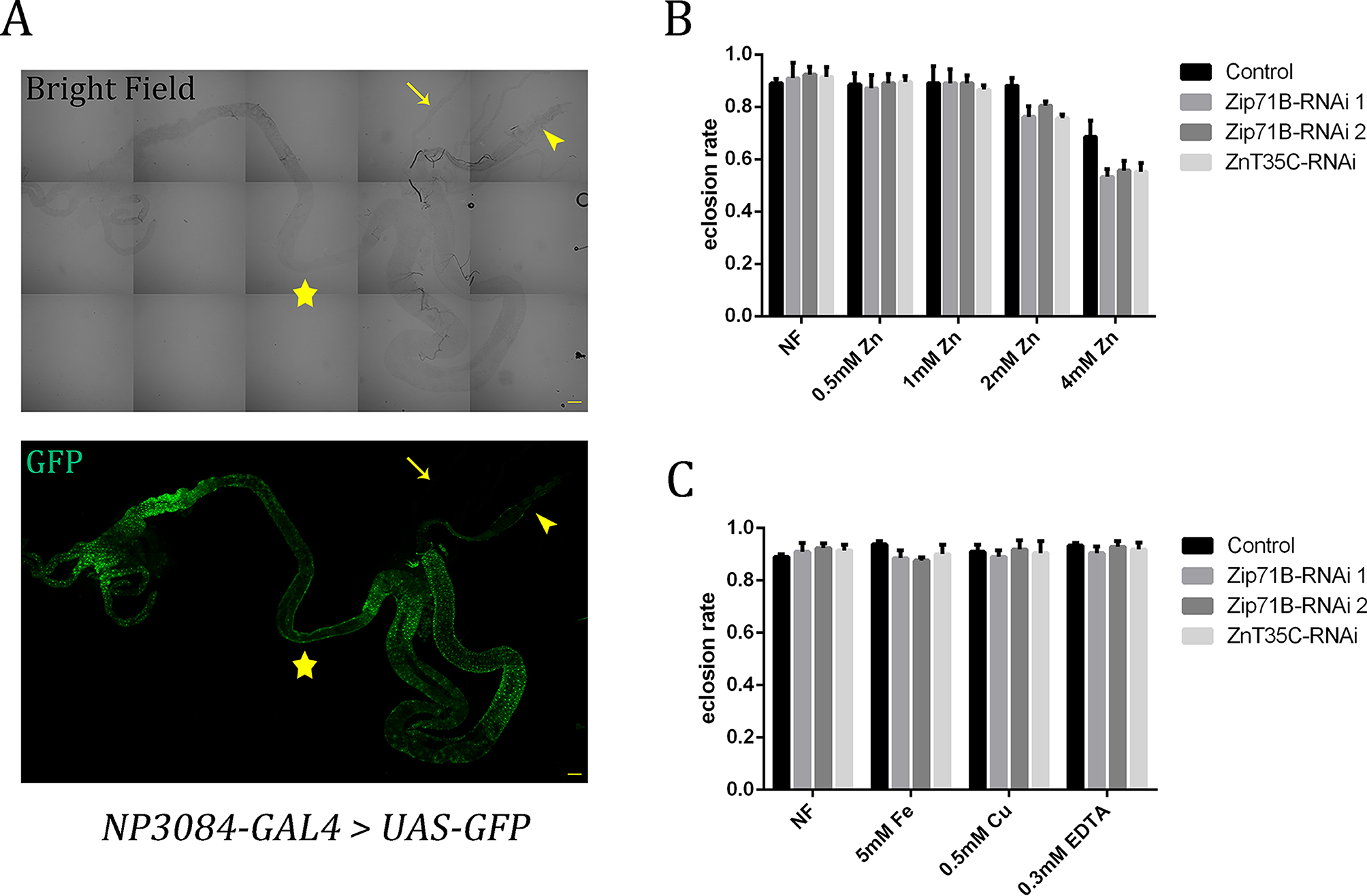

Supplement: Additional file 2: Figure S1. — Gut-specific Zip71B and ZnT35C RNAi flies display only slight sensitivity to zinc overload. (A) Expression pattern of UAS-GFP driven by NP3084-GAL4 line was visualized in dissected third-instar larvae. NP3084 directs expression all over the midgut, but not in the Malpighian tubules. The yellow arrow denotes the Malpighian tubules, arrowhead denotes the hindgut, and star denotes the midgut. Scale bars = 100 μm. (B, C) Survival rate of gut-specific Zip71B-RNAi and ZnT35C-RNAi flies under different food conditions (means ± SEM, n = 6). Genotypes of the flies are NP3084-GAL4/+ for control, Zip71B-RNAi/+; NP3084-GAL4/+ for Zip71B-RNAi, and ZnT35C-RNAi/+; NP3084-GAL4/+ for ZnT35C-RNAi. (TIF 890 kb) [file 12915_2017_355_MOESM2_ESM.tif]
